# Traditional Cox regression outperforms large language models in predicting long-term progression of intermediate to advanced hepatocellular carcinoma

**DOI:** 10.3389/fonc.2026.1710529

**Published:** 2026-01-29

**Authors:** Kang Li, Chen Wang, Yiqi Xiong, Yi Song, Yubin Zhang, Danlei Mou, Caixia Hu, Dandan Guo, Tingting Mei, Ang Li, Yonghong Zhang

**Affiliations:** 1 Biomedical Information Center, Beijing You’An Hospital, Capital Medical University, Beijing, China; 2 Interventional Therapy Center for Oncology, Beijing You’An Hospital, Capital Medical University, Beijing, China; 3 Institute of Clinical Medicine, Beijing Friendship Hospital, Capital Medical University, Beijing, China; 4 Graduate School of Dalian Medical University, Dalian Medical University, Dalian, China; 5 Department of Infectious Diseases, Beijing You’An Hospital, Capital Medical University, Beijing, China

**Keywords:** ablation therapy, Cox proportional hazards model, intermediate to advanced HCC, large language models, progression prediction

## Abstract

**Objective:**

This study aimed to evaluate and compare the performance of large language models (LLMs) and traditional Cox regression models in predicting the long-term progression risk in patients with intermediate to advanced hepatocellular carcinoma (HCC).

**Methods:**

A total of 576 patients with intermediate to advanced HCC were included, comprising a training cohort (*n* = 403) and a validation cohort (*n* = 173) for model development and validation. We evaluated the predictive performance of LLMs (DeepSeek R1, DeepSeek V3, and Qwen/QWQ-32B) and the traditional Cox regression model for estimating the progression risk of HCC at 12, 24, and 36 months. Time-dependent area under the curve (AUC), decision curve analysis, calibration curve, net reclassification improvement, and integrated discrimination improvement were used to comprehensively assess model performance.

**Results:**

Based on transarterial chemoembolization combined with targeted therapy, the addition of immune checkpoint inhibitors (ICIs) and/or ablation prolonged the progression-free survival (PFS): all four treatments combined showed optimal outcome (median PFS = 12.3 months, 95%CI = 9.9–14.1). Univariate and multivariate Cox analyses identified independent prognostic factors, which were utilized to develop a progression risk nomogram. The model had good discrimination, with training cohort AUCs (at 12, 24, and 36 months) of 0.72 (95%CI = 0.67–0.78), 0.77 (95%CI = 0.69–0.86), and 0.96 (95%CI = 0.93–0.99), respectively, and validation cohort AUCs of 0.75 (95%CI = 0.67–0.83), 0.81 (95%CI = 0.71–0.91), and 0.97 (95%CI = 0.94–1.0), respectively. Three LLMs were evaluated on the same dataset. Except for DeepSeek R1 at 12 and 24 months (training cohort), all LLMs underperformed the Cox model across time points, indicating current limitations in predicting long-term progression risk.

**Conclusion:**

The combination of ablation and/or ICIs with standard treatment could prolong PFS. In predicting the long-term HCC progression risk, the traditional Cox model exceeded the LLMs. Their combination may merge structured modeling stability with the multi-source data processing capacity of LLMs, potentially improving prediction accuracy.

## Introduction

Hepatocellular carcinoma (HCC) is one of the leading causes of morbidity and mortality worldwide (1). For intermediate to advanced HCC, transarterial chemoembolization (TACE) combined with targeted therapy is an important treatment strategy (2, 3). In recent years, immune checkpoint inhibitors (ICIs) have become a standard type of systemic therapy and have been widely used in clinical practice (4, 5). Ablation therapy has been increasingly explored in recent years for use in intermediate to advanced patients, particularly in combination with systemic treatments (5, 6). These two therapies have contributed to the development of combination therapies (7, 8). However, the variety of treatment options has made it difficult to assess prognosis on an individual basis. The Cox proportional hazards model has been extensively utilized in studies concerning HCC prognosis due to its compatibility with structured clinical variables, stable statistical properties, and strong interpretability. This model has been incorporated into clinical guidelines for risk stratification and treatment planning.

At the same time, large language models (LLMs) have shown significant potential in extracting features from unstructured data such as clinical records, imaging reports, and histopathological data (9–11). These models have demonstrated competitive performance in various diagnostic and short-term risk prediction studies (12, 13). However, the applicability of these LLMs in long-term prediction modeling remains understudied, especially in real-world settings with diverse treatment regimens and complex temporal dynamics.

In this study, we first developed a Cox model to predict the progression-free survival (PFS) in patients treated with TACE combined with targeted therapy and further stratified based on the use of ICIs and/or local ablation therapy. We then evaluated the performance of three LLMs (DeepSeek R1, DeepSeek V3, and Qwen/QWQ-32B) on the same task, comparing them in terms of discriminative ability, clinical net benefit, and risk reclassification. Our research objective was to evaluate and compare the performance of LLMs and traditional Cox regression models in predicting the long-term progression risk in patients with intermediate to advanced HCC.

## Materials and methods

### Patients

The study included 576 patients aged 18–75 years who were admitted to Beijing You’an Hospital, Capital Medical University, from January 2020 to January 2023. HCC was diagnosed according to the pathological findings or clinical–radiological features in line with the criteria of the China Liver Cancer Staging (14). Unresectability of the tumor was determined by multidisciplinary team discussion. The inclusion and exclusion criteria are described in **Supplementary Method 1**. The demographic data and clinicopathological data within 7 days before ablation were collected and are described in **Supplementary Method 2**.

### Data processing

The identical structured dataset, which was stored in CSV format and contained the same patient characteristics and outcomes, served as the basis for both the Cox model and the LLMs. The Cox model processed the data directly as a numerical matrix; for the LLM-based inference, the same CSV tables were converted into JSON Lines format. When constructing the Cox model, continuous variables [i.e., age, tumor size, and prothrombin time activity (PTA)] were discretized according to the optimal cutoff values derived from the surv_cutpoint function. Variables such as age and tumor size were not standardized as their measurement units are clinically meaningful and the scale differences among them remained within an acceptable range. For variables exhibiting wide numerical ranges [e.g., alpha-fetoprotein (AFP)], a logarithmic transformation was applied to approximate a normal distribution.

### Treatment and supplemental therapies

All enrolled patients initially received TACE combined with tyrosine kinase inhibitor (TKI) therapy. In this article, this combination therapy is referred to as “dual therapy.” The TACE procedure is described in **Supplementary Method 3**. TKI therapy was suspended for a period of 3 days prior to each TACE session and resumed 3 days later if no severe TACE-related adverse events occurred. Some patients received additional treatments (e.g., ablation therapy and/or ICIs) based on the disease characteristics, which is described in **Supplementary Method 4**.

### Follow-up

All patients were followed up every 4–8 weeks. The primary endpoint was progression, defined as new tumor lesions confirmed by imaging and/or histopathological examination after treatment, regardless of the primary or distant lesion site. PFS was defined as the time from treatment initiation to progression or death from any cause, and patients without progression and alive at the last follow-up were censored.

### Efficacy and safety assessment

Tumor response was evaluated as complete response (CR), partial response (PR), stable disease (SD), and progressive disease (PD) based on the contrast-enhanced computed tomography or magnetic resonance imaging findings according to the modified Response Evaluation Criteria in Solid Tumors (mRECIST) (15). The objective response rate (ORR) was defined as the proportion of patients who achieved the best response of CR or PR and sustained it for at least 4 weeks. The disease control rate (DCR) is the proportion of patients who achieved CR, PR, and SD. The interval between the beginning of therapy and the initial observation of CR or PR was defined as the time to response. Safety was assessed via treatment-related adverse events, which were monitored and recorded in accordance with the Common Terminology Criteria for Adverse Events version 5.0.

### Local deployment and inference environment

LLM tasks were run on a MacBook Air equipped with an Apple Silicon M4 processor and macOS 15. To enable local deployment and model inference, we utilized an open-source toolchain comprising Ollama (version 0.5.7) and LLM-Anything (version 1.7.3), described in **Supplementary Method 5**.

### Input format and prompt design

All inputs were from structured.jsonl files, with each entry representing a progression risk prediction task. Each sample included standardized clinical variables [e.g., age, tumor size, and hepatitis B virus (HBV) status, among others], formatted into natural language prompts to guide the models. Details of the task descriptions are in **Supplementary Method 6**. The task was to estimate the 12-, 24-, and 36-month HCC progression probabilities. LLMs output the progression probabilities as 0–1 decimal values. All prompts used a unified template for consistency, reproducibility, and standardization (format below).

“You are a highly experienced oncologist assessing the risk of disease progression for a cancer patient over time. Based on the provided clinical information, estimate the patient’s probability of hepatocellular carcinoma progression at 12 months, 24 months, and 36 months. You only output JSONL. Return risk of progression, a decimal score between 0 and 1. Please also provide a clear and concise explanation of your reasoning in English.”

The models were instructed to return predictions in the following structured.jsonl format: risk_12m, risk_24m, and risk_36m: Predicted progression probability at 12, 24, and 36 months; reasoning: An English explanation detailing the reasoning behind the predicted values. All inference outputs were saved in a.jsonl format to facilitate downstream structured analysis, model performance evaluation, and cross-model comparisons. During inference, all models were executed using deterministic decoding settings with temperature = 0 and top_p = 0.9, allowing for diverse yet controlled output generation. These parameters were kept constant across all runs to ensure uniform generation behavior and fair comparisons between models.

### Statistical analyses

Continuous variables are represented as the mean ± standard deviation or as median (interquartile range, IQR). The cutoff values of the quantitative variables were selected by applying the surv_cutpoint function as implemented in the “survminer” package. Risk factors were selected using univariate and multivariate Cox regression analyses and were employed to construct the final nomogram. The discrimination and the predictive accuracy were assessed using the area under the time-dependent receiver operating characteristic (ROC) curve (AUC). Consistency was evaluated using a calibration curve with Brier scores. Based on the established model, patients were stratified into the high-risk and low-risk groups according to their PFS probability. Decision curve analysis (DCA) was used to assess the clinical utility of a Cox model and the LLMs by quantifying the net benefit of using across different threshold probabilities. The net reclassification improvement (NRI) (16) was applied to calculate the proportion of correct reclassifications minus the proportion of incorrect reclassifications by the Cox model compared with the LLMs. The integrated discrimination improvement (IDI) (17) was applied to calculate the difference between the average predicted probabilities of the Cox model and the LLMs in the event and non-event groups. All statistical analyses were performed using R software (version 4.2.2) with the following packages: “rms,” “survival,” “riskRegression,” “pec,” “plotROC,” and “timeROC.” A two-sided *p*-value <0.05 was considered statistically significant.

## Results

### Characteristics of the patients

The baseline characteristics of the included patients are described in **Table 1**. A total of 576 patients with intermediate to advanced HCC at Beijing You’An Hospital were included, comprising 485 (83.6%) men and 91 (16.4%) women. The mean age of the patients was 58.39 ± 9.71 years. According to the Barcelona Clinic Liver Cancer (BCLC) staging system, 204 patients (35.4%) were classified as stage B and 372 (64.6%) as stage C. There were 126 patients (21.9%) with a single tumor, whereas 450 (78.1%) had multiple tumors. Among all patients, 98 (17.0%) received ablation only, 207 (35.9%) received ICIs only, 115 (20.0%) received both ablation and ICIs, and 155 (26.9%) received “dual therapy.” Following mRECIST, 116 (27.0%) patients achieved CR, 198 (47.0%) achieved PR, 97 (23.0%) had SD, and 11 (2.6%) had PD. The highest ORR (CR + PR) and DCR (CR + PR + SD) were 74.4% and 97.4%, respectively.

**Table 1:** Comparison of the clinical data between the training set and the validation set.

Variable	Total (*n* = 576)	Training set (*n* = 403)	Validation set (*n* = 173)	*p*
Age (years), mean ± SD	58.39 ± 9.71	58.11 ± 9.75	59.03 ± 9.63	0.30
Gender (men/women)	485/91	333/70	151/22	0.20
Ablation (yes/no)	363/213	259/144	104/69	0.39
ICIs (yes/no)	254/322	171/232	83/90	0.26
Therapy (no treatment/ablation/ICIs/ablation+ICIs)	155/98/207/115	112/58/146/87	43/40/61/29	0.39
PVTT (yes/no)	303/273	213/190	90/83	0.93
BCLC (B/C)	204/372	139/264	65/108	0.54
Tumor number (single/multiple)	126/450	97/306	33/140	0.23
Etiology (HBV/HCV/other)	472/33/71	326/19/58	146/14/13	0.03
Cirrhosis (yes/no)	486/90	449/53	142/31	0.39
Drinking (yes/no)	379/187	266/137	118/55	0.68
Smoking (yes/no)	357/219	244/159	114/59	0.26
NLR, mean ± SD	3.94 ± 3.48	3.86 ± 3.34	4.12 ± 3.8	0.26
ALT/AST, mean ± SD	1.66 ± 1.05	1.65 ± 1.04	1.67 ± 1.07	0.84
DBIL/TBIL, mean ± SD	0.44 ± 1.35	0.46 ± 1.62	0.39 ± 0.09	0.39
Child–Pugh class (A/B)	218/26	149/17	69/9	0.93
Log_10_ (AFP) (ng/ml), mean ± SD	2.13 ± 1.56	2.16 ± 1.47	2.27 ± 1.44	0.40
PTA (%), >67/≤67, mean ± SD	85.16 ± 36.83	83.18 ± 15.01	89.68 ± 62.7	0.18
mRECIST (CR/PR/SD/PD)	134/262/138/142	97/180/26/100	37/82/11/43	0.90
Tumor size(mm), >53/≤53, mean ± SD	57.69 ± 40.76	57.85 ± 41.17	57.32 ± 39.92	0.23

*ICIs*, immune checkpoint inhibitors; *PVTT*, portal vein tumor thrombus; *BCLC*, Barcelona Clinic Liver Cancer; *HBV*, hepatitis B virus; *HCV*, hepatitis C virus; *NLR*, neutrophil-to-lymphocyte ratio; *ALT*, alanine aminotransferase; *AST*, aspartate aminotransferase; *DBIL*, direct bilirubin; *TBIL*, total bilirubin; *AFP*, alpha-fetoprotein; *PTA*, prothrombin time activity; *mRECIST*, modified Response Evaluation Criteria in Solid Tumors; *CR*, complete response; *PR*, partial response; *SD*, stable disease; *PD*, progressive disease.

### Progression-free survival analysis

**Figure 1:**
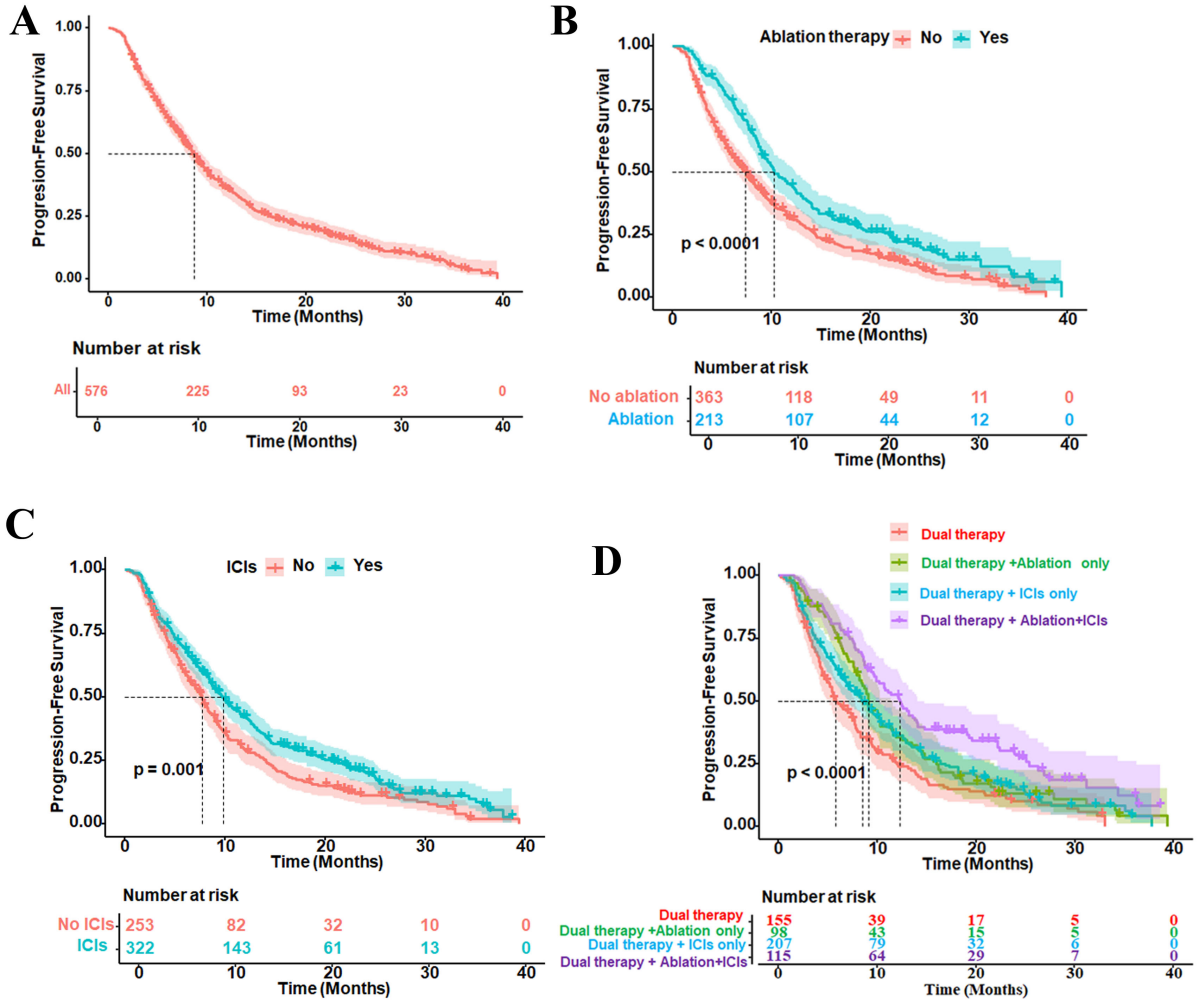
Kaplan–Meier curves for the progression-free survival (PFS) of patients with various types of therapy. **(A)** PFS of all patients, with 95% confidence intervals. **(B)** Patients who received ablation therapy. **(C)** Patients who received immune checkpoint inhibitors (ICIs) therapy. **(D)** Subgroup comparison of patients who received dual therapy [transarterial chemoembolization (TACE) combined with tyrosine kinase inhibitor (TKI) therapy]. This combination therapy was referred to as “dual therapy,” dual therapy with ablation only, dual therapy with ICIs only, or the combination of ablation, ICIs, and dual therapy. The *vertical dashed line in each panel* represents the median PFS, indicating the time at which 50% of the patients remained progression-free. *p* < 0.05 was considered statistically significant.

At the end of follow-up, the median PFS of the overall patient cohort was 8.6 months (95%CI = 7.9–9.5) (**Figure 1A**). Subgroup analysis showed that the median PFS of the patients who received “dual therapy” + ablation therapy was 10.3 months (95%CI = 9.1–12.5) (**Figure 1B**), while the median PFS of the patients who received “dual therapy” + ICI therapy was 9.8 months (95%CI = 8.6–11.5) (**Figure 1C**). Further stratification by specific treatment combinations revealed that patients who received “dual therapy” + ablation therapy combined with ICIs had the longest median PFS of 12.3 months (95%CI = 9.9–14.1), followed by the group that received “dual therapy” + ablation therapy alone at 9.1 months (95%CI = 8.2–11.6) and the group that received “dual therapy” + ICI therapy alone at 8.5 months (95%CI = 7.1–10.3). In contrast, patients who received only “dual therapy” without other interventions had the shortest median PFS of 5.8 months (95%CI = 5.1–7.7) (**Figure 1D**). These results indicate that patients with intermediate to advanced HCC have a high risk of progression. Both ablation therapy and ICI therapy helped delay the progression of HCC, and combination therapy yielded the best prognostic outcomes.

### Selection of independent prognostic factors and nomogram established for PFS

**Figure 2:**
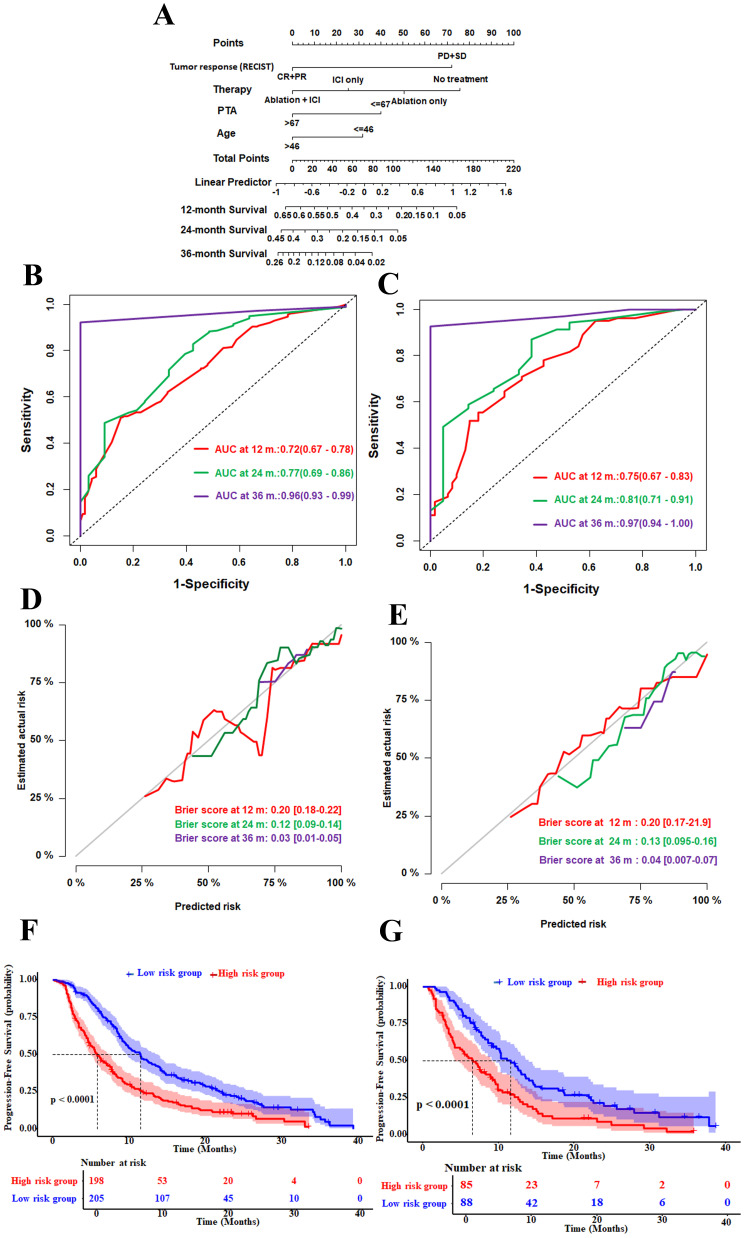
Construction and internal validation of the Cox model nomogram. **(A)** Nomogram for prediction of the 12-, 24-, and 36-month progression-free survival based on four prognostic factors. **(B, C)** Time-dependent receiver operating characteristic (ROC) curves and area under the curve (AUC) values at 12, 24, and 36 months in the training set **(B)** and the validation set **(C)**. The *numbers in the figure* represent the AUC (95% confidence interval). **(D, E)** Brier score calibration curves at 12, 24, and 36 months in the training set **(D)** and the validation set **(E)**. The *numbers in the figure* represent the Brier score (95% confidence interval). **(F, G)** Progression-free survival in the training **(F)** and validation **(G)** sets. Patients were stratified into the high- and low-risk groups based on the nomogram-derived scores. Numbers at risk are shown *below each plot*. The *p*-values were calculated using the log-rank test. The *X*-axis represents the predicted probabilities, while the *Y*-axis represents the observed probabilities.

The independent factors predictive of PFS based on univariate and multivariate Cox proportional hazards models are displayed in **Table 2**. In the univariate and multivariate analyses, age (>46 *vs*. ≤46 years), type of therapy (ablation and ICIs, ablation only, ICIs only, *vs*. no additional treatment), PTA (>67% *vs*. ≤67%), and mRECIST response (SD + PD *vs*. CR + PR) were identified as significant prognostic factors for PFS. These four independent risk factors were utilized to build a Cox model for predicting progression in patients with intermediate to advanced HCC (**Figure 2A**).

**Table 2:** Univariate and multivariate Cox regression analyses of the prognostic factors.

Variable	Univariate analysis		Multivariate analysis	
	HR (95%CI)	*p*	HR (95%CI)	*p*
Age (>46 *vs*. ≤46 years)	0.74 (0.56–0.99)	0.05	0.67 (0.50–0.91)	0.00913
Gender (men *vs*. women)	1.19 (0.92–1.53)	0.18		
Ablation (yes *vs*. no)	0.67 (0.56–0.81)	<0.001		
ICIs (yes vs. no)	0.74 (0.62–0.89)	<0.001		
Therapy				
Ablation *vs*. no treatment	0.68 (0.52–0.90)	0.006	0.77 (0.58–1.02)	0.06
ICIs *vs*. no treatment	0.74 (0.59–0.93)	0.009	0.59 (0.47–0.75)	<0.001
Ablation+ICIs *vs*. no treatment	0.48 (0.36–0.63)	<0.001	0.49 (0.37–0.65)	<0.001
PVTT (yes *vs*. no)	0.98 (0.82–1.17)	0.82		
BCLC (C *vs*. B)	0.87 (0.72–1.05)	0.15		
Tumor number (multiple *vs*. single)	1.29 (1.03–1.6)	0.02		
Etiology				
HBV *vs*. other	0.88 (0.67–1.16)	0.357		
HCV *vs*. other	0.68 (0.42–1.10)	0.118		
Cirrhosis (yes *vs*. no)	1.01 (0.79–1.3)	0.95		
Drinking (yes *vs*. no)	0.97(0.74–1.09)	0.28		
Smoking (yes *vs*. no)	0.99(0.75–1.09)	0.3		
Ascites (yes *vs*. no)	1.55 (1.17–2.06)	0.002		
NLR	1.01 (0.99–1.04)	0.28		
ALT/AST	1.14 (1.05–1.24)	0.0026		
DBIL/TBIL	1.05 (0.99–1.11)	0.08		
Child–Pugh class (B *vs*. A)	1.54 (1.04–2.29)	0.03		
Log_10_(AFP) (ng/ml)	1.09 (0.92–1.03)	0.01		
PTA (%), >67 *vs*. ≤67	0.64 (0.49–0.84)	0.0014	0.67 (0.51–0.88)	0.00454
mRECIST (SD+PD *vs*. CR+PR)	2.25 (1.85–2.73)	<0.001	2.23 (1.83–2.71)	<0.001
Tumor size (mm), >53 *vs*. ≤53	1.2 (1.0–1.44)	0.06		

*ICIs*, immune checkpoint inhibitors; *PVTT*, portal vein tumor thrombus; *BCLC*, Barcelona Clinic Liver Cancer; *HBV*, hepatitis B virus; *HCV*, hepatitis C virus; *NLR*, neutrophil-to-lymphocyte ratio; *ALT*, alanine aminotransferase; *AST*, aspartate aminotransferase; *DBIL*, direct bilirubin; *TBIL*, total bilirubin; *AFP*, alpha-fetoprotein; *PTA*, prothrombin time activity; *mRECIST*, modified Response Evaluation Criteria in Solid Tumors; *CR*, complete response; *PR*, partial response; *SD*, stable disease; *PD*, progressive disease.

The time-dependent ROC analysis showed that the AUC values for predicting progression at 12, 24, and 36 months were 0.72 (95%CI = 0.67–0.78), 0.77 (95%CI = 0.69–0.86), and 0.96 (95%CI = 0.93–0.99) in the training set, respectively, indicating that the model can effectively distinguish between progressive and non-progressive patients (**Figure 2B**). We assessed the calibration performance of the model using the Brier scores, which were 0.20 (95%CI = 0.18–0.22), 0.12 (95%CI = 0.09–0.14), and 0.03 (95%CI = 0.01–0.05) in the training set, respectively, indicating that the predicted results were highly consistent with the observed outcomes (**Figure 2D**). In the validation cohort, the nomogram also demonstrated strong discriminatory ability, with AUC values for predicting the 12-, 24-, and 36-month PFS of 0.75 (95%CI = 0.67–0.83), 0.81 (95%CI = 0.71–0.91), and 0.97 (95%CI = 0.94–1.0), respectively (**Figure 2C**). The Brier scores for progression at 12, 24, and 36 months were 0.20 (95%CI = 0.17–0.22), 0.13 (95%CI = 0.095–0.16), and 0.04 (95%CI = 0.007–0.07), respectively, further indicating that the predictive results were highly consistent with the observed outcomes (**Figure 2E**).

Patients were classified into high- and low-risk groups based on the nomogram-derived risk scores. The Kaplan–Meier curves showed that, in the training set, patients in the high-risk group had a shorter median PFS of 5.8 months (95%CI = 4.9–7.4) compared with 11.5 months (95%CI = 9.6–13.8) in the low-risk group (**Figure 2F**). In the validation set, the median PFS was 6.6 months (95%CI = 4.2–9.2) for the high-risk group and was 11.6 months (95%CI = 9.1–14.0) for the low-risk group (both *p* < 0.0001) (**Figure 2G**).

### Comparison of the predictive performance of the Cox model with that of the LLMs for tumor progression

**Figure 3:**
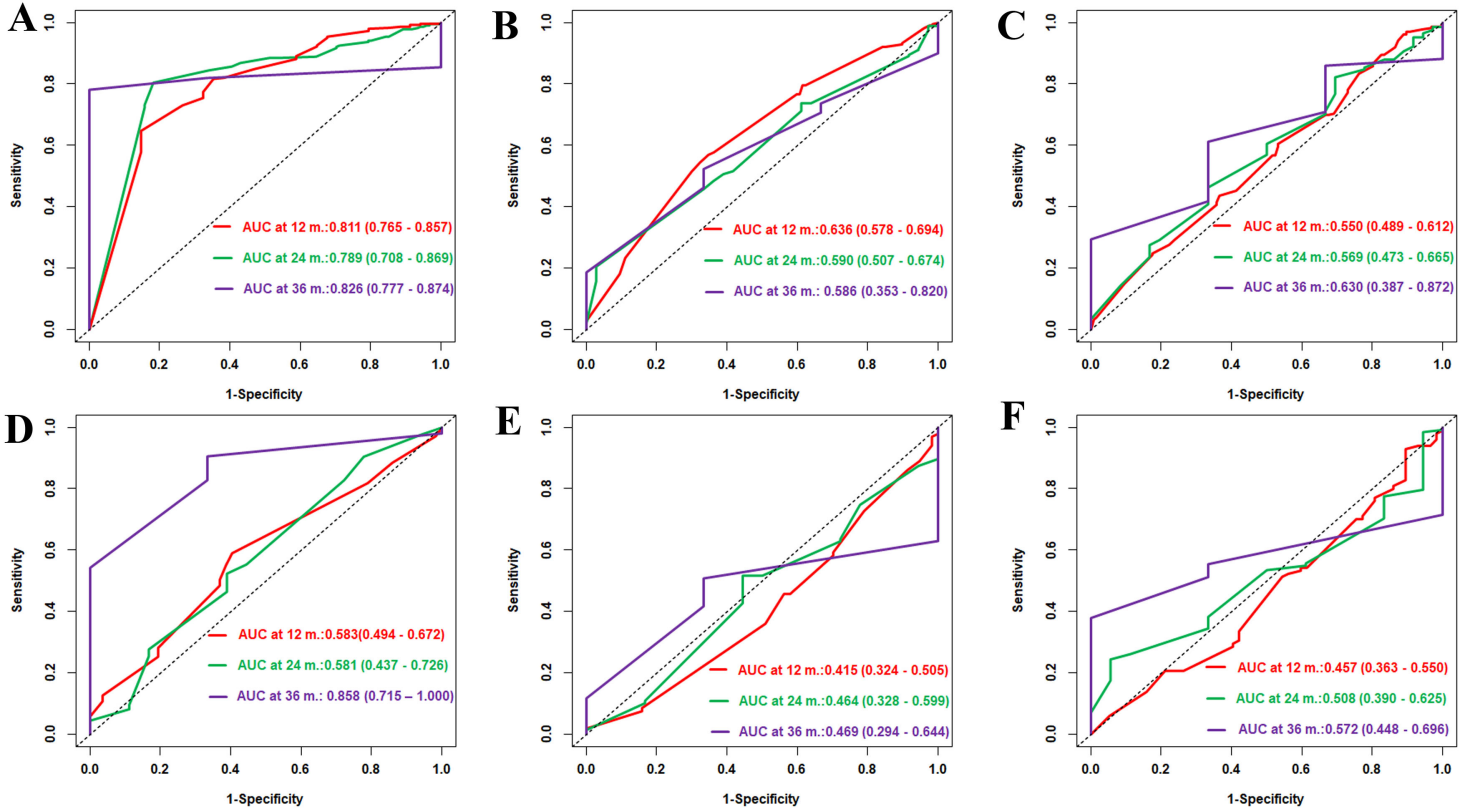
Time-dependent area under the curve (AUC) performance of DeepSeek R1, DeepSeek V3, and Qwen/QWQ-32B in the training and validation sets. **(A–C)** Time-dependent receiver operating characteristic (ROC) curves and AUCs of the DeepSeek R1, DeepSeek V3, and Qwen/QWQ-32B models at 12 months **(A)**, 24 months **(B)**, and 36 months **(C)** in the training set. **(D–F)** Time-dependent ROC curves and AUCs of the DeepSeek R1, DeepSeek V3, and Qwen/QWQ-32B models at 12 months **(D)**, 24 months **(E)**, and 36 months **(F)** in the validation set. The *numbers in the figure* represent the AUC (95% confidence interval).

**Figure 4:**
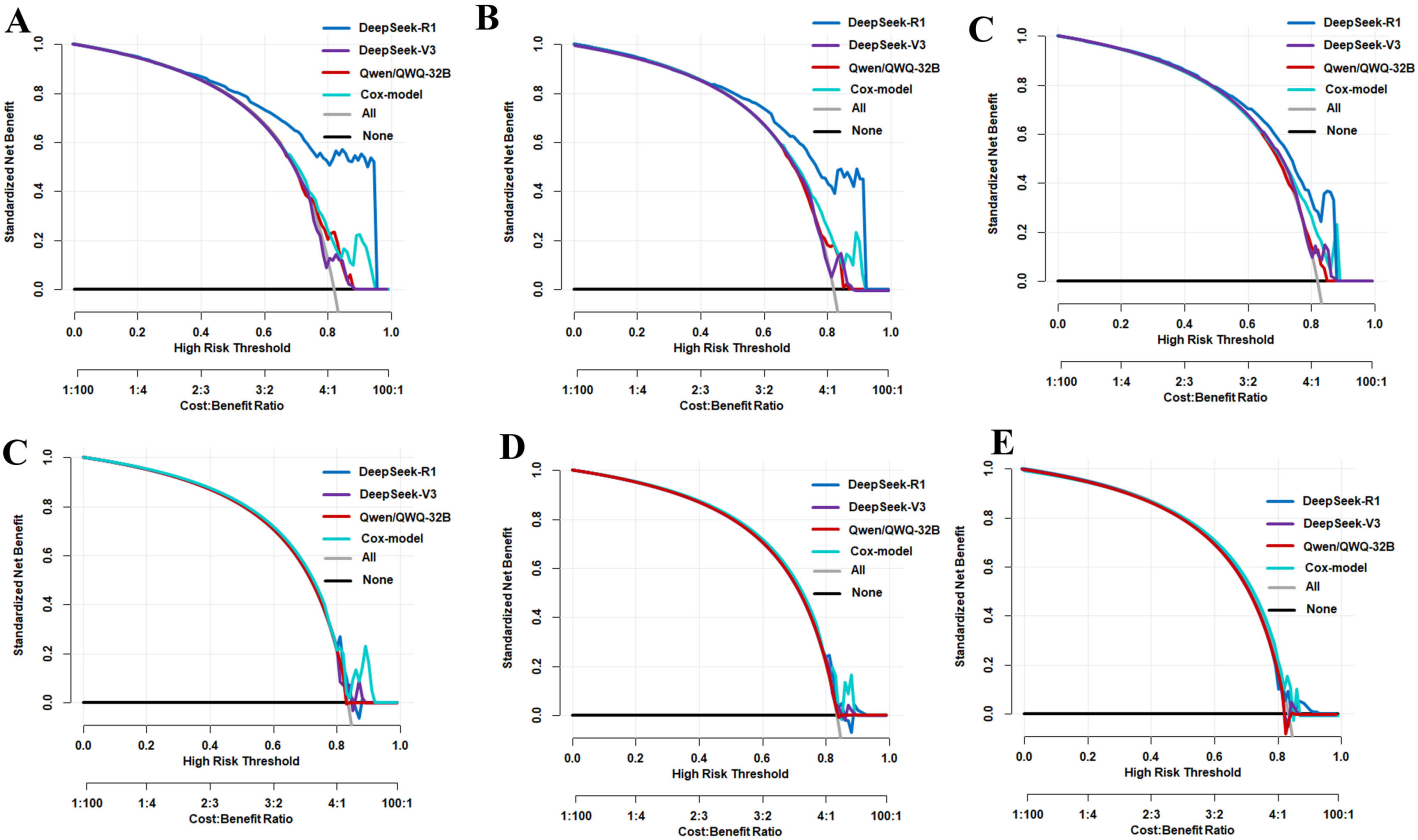
Decision curve analysis (DCA) of the predictive models at different time points in the training and validation sets. **(A–C)** Net benefit curves comparing the DeepSeek R1, DeepSeek V3, and Qwen/QWQ-32B models with the Cox model at 12 months **(A)**, 24 months **(B)**, and 36 months **(C)** in the training set. (**D–F)** Net benefit curves comparing the DeepSeek R1, DeepSeek V3, and Qwen/QWQ-32B models with the Cox model at 12 months **(D)**, 24 months **(E)**, and 36 months **(F)** in the validation set. The *X*-axis represents the high-risk threshold (predicted probability), while the *Y*-axis represents the standardized net benefit. The “*All*” and “*None*” *lines* represent the net benefit under the strategies of treating all patients or no patients, respectively.

We utilized the LLMs of the LLM-Anything platform to predict the progression risk of HCC at 12, 24, and 36 months in the training and validation cohorts. In the training set, the time-dependent ROC analysis showed that DeepSeek-R1 (**Figure 3A**) achieved AUC values of 0.811 (95%CI = 0.765–0.857), 0.789 (95%CI = 0.708–0.869), and 0.826 (95%CI = 0.777–0.874), respectively. In contrast, DeepSeek-V3 (**Figure 3B**) had lower AUC values of 0.636 (95%CI = 0.578–0.694), 0.590 (95%CI = 0.507–0.674), and 0.586 (95%CI = 0.353–0.820), while Qwen/QWQ-32B (**Figure 3C**) had AUC values of 0.550 (95%CI = 0.489–0.612), 0.569 (95%CI = 0.473–0.665), and 0.630 (95%CI = 0.387–0.872) at the same time points. In the validation set, DeepSeek-R1 (**Figure 4D**) had AUC values of 0.583 (95%CI = 0.494–0.672), 0.581 (95%CI = 0.437–0.726), and 0.858 (95%CI = 0.715–1.000), respectively. DeepSeek-V3 (**Figure 4E**) had AUC values of 0.415 (95%CI = 0.324–0.505), 0.464 (95%CI = 0.328–0.599), and 0.469 (95%CI = 0.294–0.644), while Qwen/QWQ-32B (**Figure 4F**) had AUC values of 0.457 (95%CI = 0.363–0.550), 0.508 (95%CI = 0.390–0.625), and 0.572 (95%CI = 0.448–0.696) at the same time points. Except for the performance of DeepSeek R1 at 12 and 24 months in the training set, these AUC values did not exceed the performance of the Cox model. Subsequently, we compared the DCA of DeepSeek R1, DeepSeek V3, Qwen/QWQ-32B, and the Cox model at 12, 24, and 36 months (**Figures 4A–C, respectively**) in the training cohort. DeepSeek R1 demonstrated the highest standardized net benefit at all time points, indicating superior clinical application value in predicting the progression of HCC. In contrast, DeepSeek V3 and Qwen/QWQ-32B had relatively lower net benefits, while the Cox model performed between these LLMs. In the validation cohort, the Cox model slightly outperformed the other models at 12 and 24 months (**Figures 4D, E, respectively**), while the four models performed similarly at 36 months (**Figure 4F**).

### NRI- and IDI-based performance comparison between the Cox model and the LLMs

**Figure 5:**
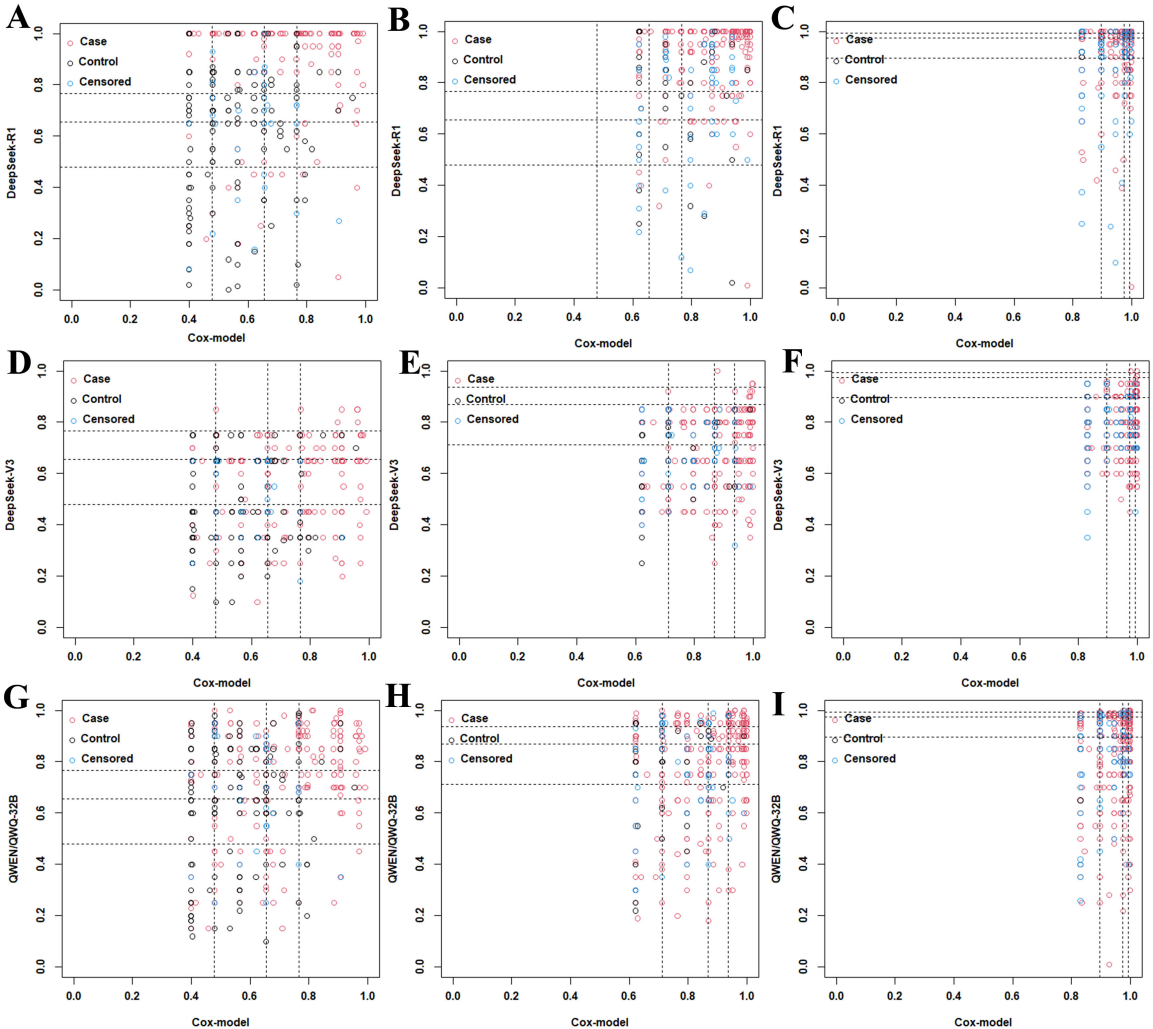
Net reclassification improvement (NRI) plots comparing the DeepSeek R1, DeepSeek V3, and Qwen/QWQ-32B models with the Cox model at 12, 24, and 36 months in the training set. **(A–C)** Category-based NRI plots for DeepSeek R1 *versus* the Cox model at 12 months **(A)**, 24 months **(B)**, and 36 months **(C)**.**(D–F)** Category-based NRI plots for DeepSeek V3 *versus* the Cox model at 12 months **(D)**, 24 months **(E)**, and 36 months **(F)**. **(G–I)** Category-based NRI plots for Qwen/QWQ-32B *versus* the Cox model at 12 months **(G)**, 24 months **(H)**, and 36 months **(I)**.

Lastly, the Cox model was used as the reference for the calculation of the NRI and IDI to assess the performance improvement of the DeepSeek R1, DeepSeek V3, and Qwen/QWQ-32B models. As shown in **Figures 5A, B** and in **Table 3**, across other time points, the Cox model consistently outperformed DeepSeek R1, DeepSeek V3, and Qwen/QWQ-32B in both the NRI and IDI metrics. DeepSeek R1 demonstrated higher NRI values than the Cox model at 12 and 24 months, with NRI values of 0.26 (0.07–0.46, *p* < 0.01) and 0.46 (0.26–0.69, *p* < 0.01), respectively. In addition, DeepSeek R1 achieved a higher IDI at 12 months compared with the Cox model, with an IDI value of 0.18 (0.09–0.27, *p* < 0.01). In the validation cohort, the Cox model consistently demonstrated superior performance compared with all LLMs (**Supplementary Figure S1**, **Table 3**), suggesting a modest improvement in overall discrimination.

**Table 3:** Comparison of the DeepSeekv3, DeepSeekr1, and Qwen models with the Cox model at 12, 24, and 36 months using net reclassification improvement (NRI) and integrated discrimination improvement (IDI).

Metric & Model Comparison	Time (months)	Training set			Validation set		
		Estimate	95%CI	*p*	Estimate	95%CI	*p*
NRI (DeepSeek-R1 *vs*. Cox model)	12	0.26	0.07–0.46	<0.01	−0.55	−0.83 to −0.28	<0.01
NRI (DeepSeek-R1 *vs*. Cox model)	24	0.46	0.26–0.69	<0.01	−0.39	−0.84 to 0.06	0.093
NRI (DeepSeek-R1 *vs*. Cox model)	36	0.04	−0.81 to 1.08	0.94	−0.28	−0.96 to 0.59	0.47
IDI (DeepSeek-R1 *vs*. Cox model)	12	0.18	0.09–0.27	<0.01	−0.08	−0.16 to −0.01	0.016
IDI (DeepSeek-R1 *vs*. Cox model)	24	−0.08	−0.17 to 0.00	0.056	−0.06	−0.15 to 0.01	0.096
IDI (DeepSeek-R1 *vs*. Cox model)	36	−0.11	−0.20 to −0.04	<0.01	−0.06	−0.22 to 0.02	0.18
NRI (DeepSeek-V3 *vs*. Cox model)	12	−0.14	−0.27 to 0.02	0.057	−0.54	−0.88 to −0.28	<0.01
NRI (DeepSeek-V3 *vs*. Cox model)	24	−0.27	−0.46 to −0.10	<0.01	−0.34	−0.75 to 0.12	0.13
NRI (DeepSeek-V3 *vs*. Cox model)	36	−0.43	−1.31 to 0.23	0.2758	0.00	−0.75 to 0.63	0.99
IDI (DeepSeek-V3 *vs*. Cox model)	12	−0.09	−0.15 to −0.03	<0.01	−0.08	−0.16 to 0.00	0.048
IDI (DeepSeek-V3 *vs*. Cox model)	24	−0.10	−0.17 to −0.03	<0.01	−0.05	−0.14 to 0.01	0.14
IDI (DeepSeek-V3 *vs*. Cox model)	36	−0.11	−0.22 to −0.02	0.016	−0.06	−0.20 to 0.02	0.25
NRI (QWEN/QWQ-32B *vs*. Cox model)	12	−0.14	−0.31 to 0.01	0.078	−0.38	−0.66 to −0.11	<0.01
NRI (QWEN/QWQ-32B *vs*. Cox model)	24	−0.29	−0.52 to −0.01	0.026	−0.08	−0.61 to 0.30	0.72
NRI (QWEN/QWQ-32B *vs*. Cox model)	36	−0.17	−1.13 to 0.51	0.69	0.29	−0.54 to 1.05	0.47
IDI (QWEN/QWQ-32B *vs*. Cox model)	12	−0.11	−0.17 to −0.05	<0.01	−0.08	−0.16 to −0.02	0.016
IDI (QWEN/QWQ-32B *vs*. Cox model)	24	−0.10	−0.18 to −0.04	<0.01	−0.05	−0.14 to 0.00	0.083
IDI (QWEN/QWQ-32B *vs*. Cox model)	36	−0.11	−0.18 to −0.03	0.016	−0.05	−0.22 to 0.01	0.2

## Discussion

In patients with intermediate to advanced HCC receiving dual therapy, the addition of ablation or ICI therapy could further prolong PFS, with the most significant effects observed when all four treatment modalities were used in combination. Existing studies have also shown that ablation therapy could improve the overall survival and PFS in patients with intermediate to advanced HCC (6, 18, 19), supporting its use as a viable combination therapy in advanced treatment strategies.

Compared with the Cox models, the LLMs required larger-scale, structurally complex multimodal data (e.g., images, text, and event logs) and substantially higher computational resources for training. A number of studies have demonstrated LLMs to show significant advantages in disease diagnosis, treatment planning, and short-term risk assessment, particularly when processing unstructured data such as clinical notes and imaging reports. One study using pathology reports from The Cancer Genome Atlas Thyroid Cancer (TCGA-THCA) cohort demonstrated that LLMs could accurately extract key information and achieve precise American Joint Committee on Cancer (AJCC) staging and American Thyroid Association (ATA) risk stratification, with F1 scores ranging from 88.5% to 96.5% for ATA risk and from 94.2% to 99.7% for AJCC staging (10). Another study on cardiac arrest patients showed ChatGPT-4 to have AUCs of 0.85 for mortality prediction and 0.83 for neurological outcome, which are comparable to those of established prognostic scoring systems (20). However, the LLMs failed to outperform the traditional Cox model, highlighting their limitations in long-term prognosis modeling in this study. A recent study supported this view: when evaluated on real-world electronic health record datasets, the predictive performance of GPT-3.5 and GPT-4 was significantly lower than that of traditional machine learning models (AUC values of 0.537 and 0.629 *vs*. 0.847 for the VUMC dataset; 0.517 and 0.602 *vs*. 0.894 for the MIMIC dataset) (21). This difference was mainly due to the LLMs not being able to resolve survival analysis issues (e.g., censored data and time-varying risks), while the Cox models were specifically designed for these situations and were more statistically robust (22–25). Majority of the LLMs only processed fixed single inputs/outputs, hardly tracking time-varying factors or multistage disease progression. Moreover, the progression of HCC is influenced by a variety of complex factors that change over time, such as the tumor biology (26, 27), pathological characteristics (28, 29), treatment efficacy (30), and liver function fluctuations (31). It is worth noting that, although the probability scores provided by the LLMs performed poorly in the quantitative evaluation, their generated reasoning text occasionally identified risk factors consistent with clinical guidelines, suggesting a potential for integrating medical knowledge. However, how to reliably extract, validate, and quantify such qualitative reasoning remains a major challenge before it can be used for serious clinical decision support. In general, LLMs are more suitable for short-term prediction tasks with clear structures and outcomes. For their application to long-term survival modeling, further optimization is needed in the model architecture and in the ability to learn from time-varying information so as to enhance their application value in clinical prognosis.

We propose Cox models and LLMs as complementary tools in survival analysis. Cox models rely on structured data with high interpretability, making them ideal for standardized clinical risk assessment (32). They remain a cornerstone of current clinical risk assessment systems. On the other hand, LLMs have excellent language comprehension and reasoning abilities, extracting insights from unstructured data (e.g., imaging reports, pathology descriptions, and clinical records) (33). They are also evolving into multimodal models capable of processing images and texts (34, 35), which help in identifying complex patterns in medical images that traditional methods might miss (33, 36). These unstructured features could be converted into structured inputs for Cox models through embedding or vectorization. If LLMs could accurately identify such features and convert them into quantifiable inputs, they could have an enhanced predictive performance without the need for specialized segmentation models. In conclusion, the Cox model could handle structured risk modeling, while the LLMs could deal with complex, unstructured, multimodal clinical data. The combination of the two could create a more versatile and interpretable hybrid clinical prediction framework fit for real-world environments with diverse and evolving clinical features. The implementation involved five key steps. Firstly, structured data (e.g., age and laboratory parameters) and unstructured text (e.g., clinical notes and imaging reports) were collected and preprocessed. Secondly, the preprocessed text was input into a clinical LLM to obtain semantic embeddings (37, 38), which were aggregated (using the [CLS] token or mean pooling) and then dimensionally reduced via principal component analysis. Thirdly, the resulting text-derived features were concatenated with the structured variables to form a unified feature vector. Fourthly, this combined feature set, along with the survival time and the event status, was used to train the Cox model. Finally, model performance was evaluated using the C-index, AUC, calibration curves, and DCA.

This study also has certain limitations. Firstly, it was based on retrospective data from a single center (576 patients) and did not undergo external validation in a multicenter independent cohort; therefore, the robustness of its conclusions needs further confirmation. Secondly, the exceptionally high AUC values observed at 36 months for the Cox model (0.96 in training and 0.97 in validation) should be interpreted with caution as they are likely attributable to the extreme class imbalance at this late time point, with only three patients remaining event-free in each cohort. Such a small number of non-events made the discrimination metric estimates unstable. Consequently, the model performance at time points with a more sufficient sample size, such as at 24 months [AUC: training = 0.77 (95%CI = 0.69–0.86), validation = 0.81 (95%CI = 0.71–0.91)], provided a more robust and representative assessment of its generalizability. Finally, the evaluation of the LLMs relied solely on structured clinical data, without incorporating unstructured information (e.g., imaging, pathology, or genomics), which might have underestimated their potential predictive value.

## Conclusions

In summary, combining ablation or ICIs with standard treatment significantly prolonged the PFS in patients with intermediate to advanced HCC, with dual therapy showing superior efficacy. In addition, the constructed Cox regression model effectively distinguished between the high-risk and low-risk patients with advanced progression, and its predictive performance was superior to that of three LLMs. The combination of Cox and LLMs is expected to integrate the robustness of structured models with the ability of LLMs to process multi-source heterogeneous data, thereby achieving higher prediction accuracy.

## Data Availability

The original contributions presented in the study are included in the article/**Supplementary Material**. Further inquiries can be directed to the corresponding author.
