# Supplementary material for: Traditional Cox regression outperforms large language models in predicting long-term progression of intermediate to advanced hepatocellular carcinoma
Source: Front Oncol. 2026 Jan 29;16:1710529. doi: 10.3389/fonc.2026.1710529 (PMC12893998; doi:10.3389/fonc.2026.1710529)
Supplement: Supplementary file 1 [file DataSheet1.docx]

Supplementary Material

# Supplementary Method

**Supplementary Method 1:** **The inclusion and exclusion criteria**

The inclusion criteria were: (1) age 18-75 years, (2) patients with intermediate to advanced HCC who received TACE combined TKI therapy, and (3) at least one measurable lesion in accordance with the mRECIST. The exclusion criteria were: (1) Child-Pugh class C, (2) Eastern Cooperative Oncology Group performance status score greater than 1, (3) combined with other malignancies, (4) auto- immune disease that is being treated or immunodeficiency, and (5) missing important data. In order to establish a more reliable and robust model, the patients included were randomly divided into a training set (N1 = 403) and a validation set (N2 = 173), and the demographic characteristics, laboratory data and prognosis of patients in the two groups were compared. Informed consent was exempted by the ethics committee because it was a minimum-risk study.

**Supplementary Method 2: The clinicopathological data collection**

Demographic data included age, gender, and history of smoking, drinking, antiviral, hypertension and diabetes mellitus. Clinicopathological data included following: (1) tumor information: number, size, and alpha-fetoprotein;(2) liver function indicators: cirrhosis; (3) laboratory parameters: neutrophils, platelets, lymphocytes, alanine aminotransferase, aspartate aminotransferase, total bilirubin, albumin, prothrombin time activity (PTA), g-glutamyl transpeptidase, alkaline phosphatase, international normalized ratio; (4) Treatment-related factors: details of TACE and the use of targeted therapy, ICIs, and ablation.

**Supplementary Method 3: The TACE procedure**

TACE procedure: using the Seldinger technique, the right femoral artery was punctured while the patient was under local anesthesia. Subsequently, angiography of the common hepatic artery or celiac artery was conducted to identify the number, position, size, and supplying arteries of the target lesions. A microcatheter was super-selectively inserted into the tumor-supplying arteries, followed by the infusion of a mixed emulsion of iodide oil (5-20 mL) and pirarubicin. Ultimately, the blood flow in the supplying arteries was interrupted by selective embolization using gelatin sponge granules. Depending on the evaluation of the target tumor (especially evidence of arterial blood supply) and hepatic function, TACE was repeatedly conducted every 4-6 weeks on demand. TKI included sorafenib, lenvatinib, regorafenib, donafenib, or cabozantinib and initiated within 3-14 days after TACE treatment. The choice of TKI was determined by the attending physician according to patient tolerance and clinical guidelines. For patients receiving TKI (body weight < 60 kg: 8 mg; body weight ≥ 60 kg: 12 mg), the drug was administered orally once daily.

**Supplementary Method 4:** Supplemental Therapies

**Ablation Therapy**

In this study, a subset of patients underwent additional locoregional ablation therapy based on tumor burden, lesion location, and liver function reserve. Notably, ablation was not administered with curative intent, but rather served as a palliative measure within a multimodal treatment strategy aimed at enhancing local tumor control and delaying progression in selected patients with intermediate to advanced HCC. The decision to perform ablation was made by a multidisciplinary tumor board.

**Immune Checkpoint Inhibitors**

PD-1 inhibitors (camrelizumab 200 mg, tislelizumab 200 mg, or sintilimab 200 mg) were administered intravenously every 3 weeks. The same peri-procedural withholding protocol as TKI was applied during TACE sessions.

**Supplementary Method 5:** Local deployment and inference environment

Ollama leverages the integrated Apple GPU to achieve efficient on-device inference, enabling LLM to run without relying on external GPUs or cloud services. LLM-Anything is used to batch-process structured prompts from. jsonl input files by calling the local API interface provided by Ollama. All inference tasks are executed in an offline environment, ensuring data privacy and complete independence from internet resources. This study employed three categories of open-source large language models (LLMs) developed by AI research institutions in China: the DeepSeek-R1 and DeepSeek-V3 models released by DeepSeek, and the Qwen-32B model developed by Alibaba Group.

**Supplementary Method 6: Detailed task description for the large language models**

**Dataset Overview:** You are a highly experienced oncologist assessing the risk of disease progression for a cancer patient over time. Based on the provided clinical information, estimate the patient's probability of Hepatocellular Carcinoma progression at 12 months, 24 months, and 36 months. You only output JSONL. Return Risk of progression, a decimal score between 0 and 1. Please also provide a clear and concise explanation of your reasoning in English.

**Patient Identification**: De-identified patient ID

**Demographics:** Sex: (1=Male，2=Female); Age: Patient age (in years)

**Disease Evaluation and Outcomes**

RECIST: Response Evaluation Criteria in Solid Tumors (CR, PR, SD, PD)

P.status: Whether the patient experienced tumor progression (Disease 0=Not Progressed，1=Disease Progressed)

P.month: Time without disease progression (in months)

**Treatment Information:**

Immune: Whether the patient received ICI therapy (1=Received ICI treatment;0=No ICI treatment).

Ablation:Whether the patient received ablation therapy (1 = received ablation therapy, 0 = did not receive ablation therapy)

Combination therapy: Includes four treatment types

0 = neither ablation nor immunotherapy

1 = ablation only

2 = immunotherapy only

3 = both ablation and immunotherapy

Tumor Characteristics:

PVTT: Presence of portal vein tumor thrombus (1 = PVTT present, 0 = PVTT absent)

Metastasis: Presence of metastasis (1 = metastasis present, 0 = no metastasis)

BCLC: Barcelona Clinic Liver Cancer (BCLC) stage (2 = stage B, 3 = stage C)

Tumor number: Tumor number grouping (1 = multiple tumors, 0 = single tumor)

TumorSize: Maximum tumor diameter (in mm)

**Clinical History:**

**Etiology:** Etiology (1 = chronic hepatitis B, 2 = non-chronic hepatitis B)

**Cirrhosis:** Liver cirrhosis (1 = cirrhosis present, 0 = no cirrhosis)

**Drinking:** Alcohol consumption (1 = drinking, 0 = non-drinking)

**Smoking:** Smoking history (1 = smoker, 0 = non-smoker)

**Laboratory Test Results**

**NLR:** Neutrophil-to-lymphocyte ratio

ALT/AST: ALT/AST ratio

DBIL/TBIL: DBIL/TBIL ratio

Child.Pugh: Child–Pugh score (1 = Class A, 2 = Class B)

PTA: Prothrombin activity (%)

AFP: Alpha-fetoprotein

# Supplementary Figures and Tables

## Supplementary Figures


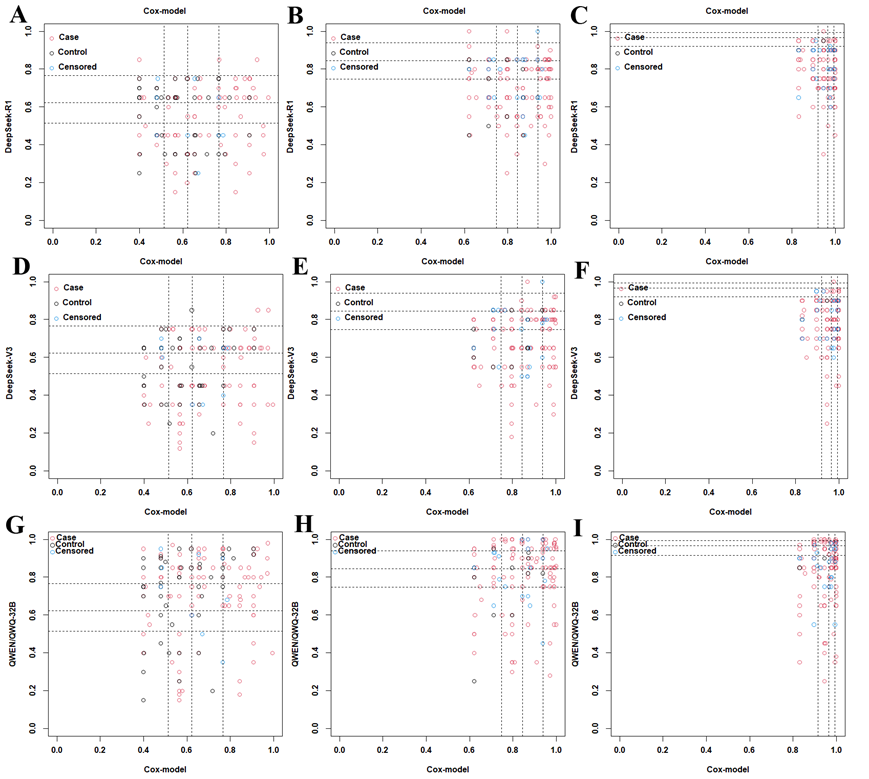


**Supplementary Figure 1. NRI plots comparing DeepSeek R1, DeepSeek V3, and Qwen/QWQ-32B models with the Cox model at 12, 24, and 36 months in the validation set.** Category-based NRI plots for DeepSeek R1 versus the Cox model at 12 months (A), 24 months (B), and 36 months (C). Category-based NRI plots for DeepSeek V3 versus the Cox model at 12 months (D), 24 months (E), and 36 months (F). Category-based NRI plots for Qwen/QWQ-32B versus the Cox model at 12 months (G), 24 months (H), and 36 months (I). Abbreviations: NRI, net reclassification improvement.
